# Group Acceptance and Commitment Therapy for Recovery From Psychosis: Protocol for a Single-Group Waitlist Trial

**DOI:** 10.2196/49849

**Published:** 2024-03-18

**Authors:** Marilyn L Cugnetto, Eric M J Morris, Siobain F Bonfield, Jesse Gates, Ilona Morrison, Ellie R Newman, Julia D Nicholls, Lisa M Soares, Megan T Antonucci, Jacinta R Clemente, Claire L M Garratt, Eliot Goldstone, David A Pavone, John Farhall

**Affiliations:** 1 Mental Health Division Northern Health Epping, Victoria Australia; 2 Department of Psychology, Counselling & Therapy School of Psychology & Public Health La Trobe University Bundoora, Victoria Australia; 3 Peninsula Health Mental Health Service Frankston, Victoria Australia; 4 Orygen Parkville, Victoria Australia; 5 St Vincent’s Hospital Melbourne, Victoria Australia; 6 Alfred Mental and Addiction Health Melbourne, Victoria Australia; 7 NorthWestern Mental Health Royal Melbourne Hospital Melbourne, Victoria Australia

**Keywords:** Acceptance and Commitment Therapy, ACT, group therapy, interventions, personal recovery, protocol, psychosis, psychotic disorders, public mental health services

## Abstract

**Background:**

Psychological interventions, along with antipsychotic medications, are recommended for adults diagnosed with a psychotic disorder. While initially designed to mitigate positive symptoms, psychological interventions targeting personal recovery were developed and aligned with the recovery framework that many mental health services have adopted. Acceptance and Commitment Therapy (ACT) for psychosis is one such intervention that shows promise when delivered in an individual format. There is preliminary evidence that ACT for psychosis in a group format improves recovery.

**Objective:**

This trial aims to evaluate the effectiveness of the “Recovery ACT” group program on personal recovery among adults living with a psychotic disorder.

**Methods:**

Our unfunded study is a multiagency, prospective, nonrandomized, waitlist control, single-group trial of the Recovery ACT group program. The program involves 7 weekly group sessions of 90 minutes duration and a 90-minute booster session held 1 month later. We intend to recruit 160 adults living with a psychotic disorder who enroll in a group that is offered as a routine clinical service at participating public mental health services in Melbourne, Victoria, Australia. The 4 assessment time points are 4-6 weeks before the start of the group program, at the start of the group program, at the end of the group program, and at the booster session. There is an optional midgroup assessment and follow-up study. The primary outcome is personal recovery. Secondary outcomes include participants’ well-being and psychological flexibility processes. Qualitative data are also collected from participants and facilitators.

**Results:**

Recruitment began in September 2019 and is ongoing until 2024, subsequent to a 24-month disruption due to the COVID-19 pandemic. As of the submission of this paper, 93 participants consented to the evaluation, 65 completed T1 measures, and 40 had a complete data set for the proposed analyses.

**Conclusions:**

This is the first trial evaluating the effectiveness of the Recovery ACT group program on personal recovery for adults living with a psychotic disorder. Findings will contribute to knowledge about psychosocial interventions for adults living with psychosis. This trial may also serve as an example of a partnership between clinicians and academics that can facilitate the translation of research into practice.

**Trial Registration:**

Australian New Zealand Clinical Trials Registry ACTRN12620000223932; https://anzctr.org.au/Trial/Registration/TrialReview.aspx?ACTRN=12620000223932

**International Registered Report Identifier (IRRID):**

DERR1-10.2196/49849

## Introduction

### Background and Rationale

Schizophrenia spectrum disorders have a prevalence rate of 3.3 per 1000 individuals worldwide [1], with schizophrenia ranking as the twelfth most disabling disorder [2]. Psychotic disorders are typically first diagnosed in young adulthood and can run a chronic course [3]. About two-thirds of individuals diagnosed with a psychotic disorder continue to experience persistent psychotic symptoms despite adhering to antipsychotic medication regimens; about 20% do not respond at all [4]. In addition, there is a high prevalence of comorbid anxiety and mood disorders—about 40% experience anxiety [5,6] and 30%-80% experience depression [6,7]—and other challenges (eg, stigma and social exclusion) that impact well-being and functioning. Persisting psychotic symptoms, comorbid anxiety and depression, and other challenges not only contribute to an individual’s level of distress and functioning but also impact a person’s ability to fully engage in life [8], referred to as personal recovery [9].

For over 2 decades, psychological and psychosocial interventions have been internationally recommended as adjuncts to pharmacological interventions [10-13]. The leading evidence-based psychological intervention recommended for psychotic disorders is cognitive behavioral therapy for psychosis (CBTp). This intervention involves teaching skills to modify unhelpful thinking patterns that impact behavior [14] and primarily targets reduction in psychotic symptoms and, arguably, distress and functioning as well. While meta-analyses demonstrate that CBTp reduces psychotic symptoms (most notably positive symptoms) with a small effect size [15-17], CBTp does not appear to mitigate distress or improve quality of life [18]. The latter findings continue to be challenged [19,20]. Modifications of CBTp (eg, low-intensity CBTp [21,22] and group CBTp [23]) are promising, though they have yet to amass robust evidence to be recommended in treatment guidelines.

Another promising psychological therapy for psychotic disorders is Acceptance and Commitment Therapy (ACT). This approach involves developing psychological flexibility, which is the ability to be in conscious contact with the present moment, and the capacity to persist with or change behavior based on whether it aligns with one's personal values [24]. Psychological flexibility processes include willingness to be with mental experiences (thoughts, feelings, sensations, etc) instead of engaging in experiential avoidance; cognitive defusion (distancing or observing with openness and curiosity) with mental experiences; engagement in valued actions; and mindfulness (ie, awareness of the present moment) [24]. ACT adapted for individuals living with a psychotic disorder has been implemented in both inpatient and outpatient settings. There is emerging evidence [25] that this intervention not only reduces rehospitalization [26-28] but can also improve quality of life and personal recovery [29].

The first ACT for psychosis group intervention targeting quality of life and social functioning was a 4-session group (2-hour sessions run weekly) known as the “ACT for Life” program, a manualized protocol developed by researchers in the United Kingdom for adults living with psychosis [30,31]. An initial uncontrolled study evaluating this program at a community psychosis service in the United Kingdom [32] demonstrated small improvements in functioning and mood after group completion. A subsequent controlled study [33] demonstrated the positive effects of group participation on well-being over time and greater independence, as indicated by service use changes. Both studies demonstrated that the group was feasible and acceptable to adults living with psychosis.

Building on promising results from individual and group ACT programs for psychosis, the ACT for Life program was adapted for public outpatient mental health services in Melbourne, Victoria, Australia [34]. A pilot of the adapted program, referred to as “Recovery ACT,” was implemented by public mental health service clinicians as part of their routine practice and evaluated using pre- and postgroup outcome measures together with qualitative feedback from facilitators and participating consumers [35]. Results from the uncontrolled pilot study involving 9 groups and 90 consumers demonstrated that the program and its evaluation were feasible, acceptable, and safe [35]. Significant increases in personal recovery, well-being, and psychological flexibility were observed from the start to the end of the program. The extent of consumer and clinician interest in the groups, both from the original agency and additional agencies, strongly indicated the feasibility of conducting a more extensive evaluation. Building on this momentum, our clinician-academic partnership agreed to conduct this more stringent trial by incorporating a waitlist control period and an established measure of personal recovery as the primary outcome.

### Objectives

The primary aim is to evaluate the effectiveness of the Recovery ACT group program on personal recovery for adults living with a psychotic disorder in routine clinical practice across public mental health organizations in Australia using a single-group, waitlist control design. A secondary aim is to evaluate the effect of the Recovery ACT group program on well-being and psychological flexibility processes (experiential avoidance, cognitive defusion, engagement in valued actions, and mindfulness). The research questions are as follows: (1) What are the clinical benefits and risks of the Recovery ACT group program for adults diagnosed with psychotic disorders as conducted in routine practice in Australia? (2) Are there changes in psychological flexibility processes due to participation in the Recovery ACT group program? (3) Are changes in psychological flexibility processes associated with changes in personal recovery and well-being?

The primary and secondary hypotheses are that the longitudinal trajectory of personal recovery and the secondary outcomes will not significantly change over the waitlist period, then will improve from the start to the end of the group program, and then improve at a less rapid rate from the end of the group to the booster session. A further hypothesis is that changes in psychological flexibility processes during the first half of the group program will be associated with changes in personal recovery and well-being in the second half of the group program.

## Methods

### Participants and Study Setting

We plan to recruit a minimum of 160 adults (aged between 18 and 65 years) who enroll in a Recovery ACT group program at 1 of 4 public mental health service study sites in Melbourne (NorthWestern Mental Health, St Vincent’s Mental Health Service, Alfred Mental and Addiction Health, and Peninsula Health Mental Health Service). The target minimum number of participants at NorthWestern Mental Health is 95 participants, and 32 participants each at the other study sites. The overall recruitment target was based on the minimum sample size needed to complete the intended analyses and an anticipated 60% participant retention rate based on the pilot study [35].

The inclusion criteria are a primary file diagnosis of either schizophrenia spectrum disorder or other psychotic disorder, a mood disorder with psychotic features, or a substance-induced psychotic disorder; currently receiving outpatient mental health care at 1 of the study sites; and current enrollment in a Recovery ACT group program. The exclusion criteria are lack of capacity to consent to the evaluation; lack of proficiency in speaking and comprehending English; a file diagnosis of intellectual disability or borderline personality disorder.

### Study Design

**Figure 1:**
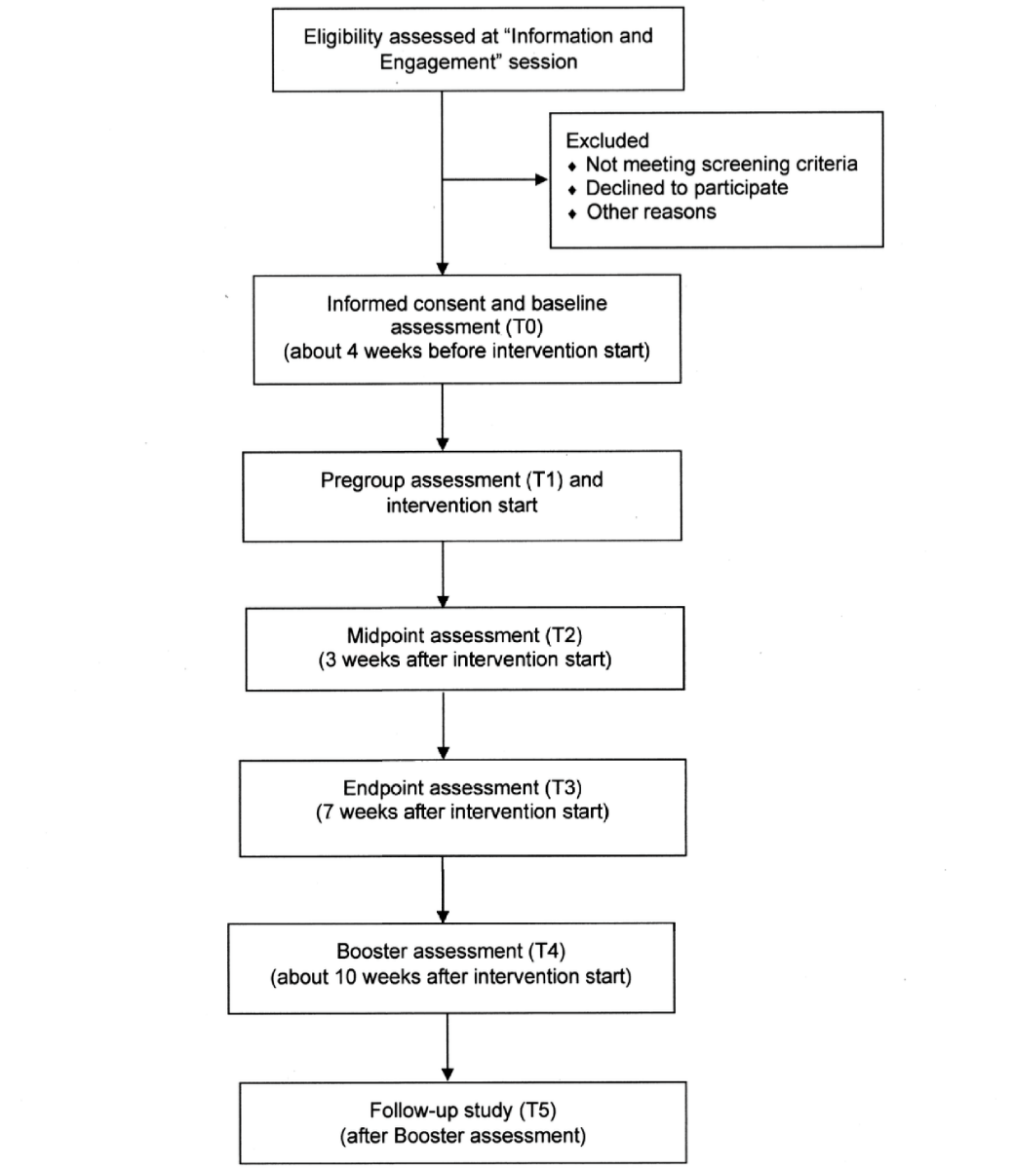
CONSORT (Consolidated Standards of Reporting Trials) diagram of the Recovery ACT Trial. ACT: Acceptance and Commitment Therapy.

This trial is a multiagency, prospective, longitudinal, nonrandomized, waitlist control, single-group study among Australian adults diagnosed with a psychotic disorder who participate in a routine Recovery ACT group program. The design includes 4 assessment time points: the start of the waitlist period (approximately 4-6 weeks before the start of the group program), the start of the group program (0 weeks), the end of the group program (about 6 weeks after the start of the group program), and the booster session (about 10 weeks after the start of the group program). Some participants may complete outcome measures at the midpoint session (T2, which is 3 weeks after the start of the group program) if the study site is resourced to collect data at an additional time point. Quantitative data from facilitators about participants’ clinical severity and clinical improvement will be collected before, during, and after the group program, as well as relevant demographic and clinical information collected at baseline from participants’ electronic medical records. At either the end of session 7 or the booster session, participants will be asked to provide written consent for study personnel to contact them for a follow-up study. Facilitators will be invited to complete a semistructured interview with senior investigators after each group program to gather qualitative data about the feasibility of the group program in routine practice and to assist in the interpretation of the quantitative measures completed by participants. The protocol development addressed the SPIRIT (Standard Protocol Items: Recommendations for Interventional Trials; Multimedia Appendix 1) guidelines [36] and intends to adhere to the CONSORT (Consolidated Standards of Reporting Trials; Figure 1) criteria [37]. We retrospectively registered the trial with the Australian New Zealand Clinical Trials Registry; Table 1 lists the trial registration data set.

**Table 1:** World Health Organization Trial registration data set.

Data category		Information
Primary registry and trial identifying number		ANZCTR, ACTRN12620000223932
Date of registration in primary registry		February 24, 2020
Secondary identifying numbers		UTN: U1111-1247-8144
Source of monetary or material support		Royal Melbourne Hospital, NorthWestern Mental Health
Primary sponsor		Royal Melbourne Hospital, NorthWestern Mental Health
Secondary sponsor		La Trobe University
Contact for public enquiries		Dr Eric Morris
Contact for scientific enquiries		Dr Marilyn Cugnetto
Public title		Acceptance and Commitment Therapy (ACT) Group Program for Recovery from Psychosis: A Multi-Agency Evaluation
Scientific title		A Multi-agency Evaluation of the Routine Practice of an Acceptance and Commitment Therapy Group for Recovery from Psychosis
Countries of recruitment		Australia
Heath conditions or problems studied		Psychosis, Mental Health, Schizophrenia
Interventions		Treatment: “Recovery ACT for Psychosis” Group Program; comparator or control: single group, waitlist control period
**Key inclusion and exclusion criteria**		
	Inclusion criteria	Inclusion criteria: Out-patients aged between 18 and 65 years; have a primary file diagnosis of either a schizophrenia spectrum or other psychotic disorder, a mood disorder with psychotic features, or a substance-induced psychotic disorder; are currently receiving outpatient mental health care at one of the study centers; are self-referred or referred by clinical staff to a Recovery ACT group being conducted by a participating center.
	Exclusion criteria	Exclusion criteria: lack of capacity to consent to this evaluation; lack of proficiency in speaking and comprehending English; file diagnosis of an intellectual disability (an intellectual development disorder) or borderline personality disorder.
Study type		Interventional
Allocation		Nonrandomized trial Intervention assignment: single-group Masking: open (masking not used)
Primary purpose		Treatment
Phase		N/A^a^
Date of first enrollment		September 13, 2019
Target sample size		160
Recruitment status		Recruiting
Primary outcomes		Personal recovery
Key secondary outcomes		Well-being

^a^N/A: not applicable.

### Procedure

#### Recruitment

Participants will be recruited from current consumers of the services enrolled in a Recovery ACT group program as part of their normal clinical care. Recovery ACT group programs are conducted at all 4 participating sites, either as part of a program of groups offered to consumers or as periodic opportunities. All sites follow the same recruitment plan. Recruitment will continue until the target is met, as long as the clinical services are willing to continue to support the evaluation. Facilitators will describe the group program and the evaluation study at staff meetings and with fellow clinicians at their study site to ensure they are aware that an evaluation of the program is occurring and that consumers will be invited but not obligated to participate. Facilitators will receive all referrals to the group program and contact potential consumers to schedule an “Information and Engagement” session to brief consumers about the group program and confirm their wish to attend. All consumers who enroll in the group program will be screened for eligibility to participate in the evaluation. Consumers who meet the eligibility criteria will be provided with an explanation of the study, an information sheet, and an evaluation brochure, and those who choose to participate will be required to provide written informed consent.

After the completion of a group program (after the booster session), senior investigators will invite facilitators to participate in a semistructured group interview for evaluation purposes and seek informed consent.

Due to the time elapsed from the initial consent process, all group members will be asked again at either session 7 or the booster session for permission and written consent to contact them in the future to invite them to participate in a follow-up study on the experience of participating in the group.

#### Intervention

The Recovery ACT group program includes 7 core sessions scheduled weekly and a booster session scheduled approximately 4 weeks later. The duration of all sessions is 90 minutes, and they are delivered in person. The intervention is an adaptation of the ACT for Life program [30] that centers on a core metaphor (passengers on the bus) [38] to facilitate consumers’ engagement in value-based actions when relating to their internal experiences (eg, thoughts, feelings, and memories) once they become aware of the internal experiences and are able to defuse (or cognitive distance) from them. The group focuses on developing awareness of internal experiences through metaphors and mindfulness activities and building defusion skills. In the metaphor, the “bus” represents the consumer’s life in the present moment, the “bus driver” is the consumer, and the “passengers” on the bus are the consumer’s internal experiences. The topics covered during the core sessions include: introduction to noticing (mindfulness) of present experiences and values; enacting the core passengers on the bus metaphor and “specific, meaningful, adaptive, realistic, and time-framed” (SMART) goals; review of values, noticing, and willingness; identification of passengers and different ways of responding to them and development of noticing skills; passengers on the bus and changing relationships with words; review of values and encouragement of sharing value-guided actions; and review of key group messages. Brief contact with consumers between sessions is encouraged to promote completion of home practice and to encourage attendance.

There are no specified criteria for discontinuing or modifying the intervention, as this is an evaluation of a group program as conducted in routine clinical practice at local mental health services. Participants can choose to withdraw from the evaluation and remain in the group program. Participants may be withdrawn from the study due to a protocol violation or an adverse event.

Facilitators will be clinical mental health staff employed at a participating public mental health service who, where possible, have participated in an introductory ACT workshop and completed training in Recovery ACT. The group is led by 2 facilitators, one of whom is experienced (ie, has previously led a Recovery ACT group program) and adheres to the Recovery ACT group program manual [39]. Facilitators will be invited to attend a minimum of 3 “Support and Supervision” sessions while facilitating a group. The goal of these sessions is to enhance fidelity to the Recovery ACT group program manual [39]. In addition, facilitators will complete a fidelity log after each group session.

There are no restrictions on concomitant care and interventions, as this is an unfunded evaluation of the Recovery ACT group program that is already offered in routine clinical care at the participating public mental health services. To participate in the Recovery ACT program, participants must be receiving care at the respective public mental health service. As such, participants will receive provisions for ancillary and posttrial care at the public mental health service. The potential risks related to the study are deemed minimal. Facilitators are responsible for participants’ clinical care during the group program, including distress related to study procedures. Participants have a key support clinician for out-of-group issues that may arise.

### Outcomes

#### Timeline

**Figure 2:**
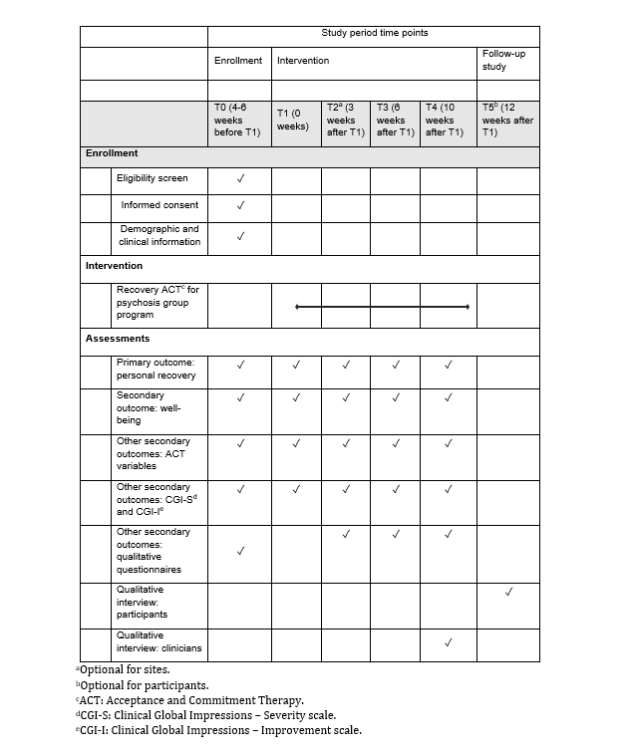
Schedule of enrollment, interventions, and assessments.

The schedule of enrollment, interventions, and assessments is displayed in Figure 2. The quantitative self-report outcome measures are intended to be completed independently at the start of the waitlist period (T0, approximately 4-6 weeks before the start of the group program, typically at the “Information and Engagement” session), at the start of the group program (T1), at the end of the group program (T3, which is 6 weeks after the start of the group program), and at the booster session (T4, about 10 weeks after the start of the group program). Some participants may complete outcome measures at the midpoint session (T2, which is 3 weeks after the start of the group program) if the study site is resourced to collect data at an additional time point. Measures are completed after the group session, except for at T1, when participants complete measures before the start of the group to ensure no exposure to the program’s content. Participants can request assistance from facilitators to complete the measures. Demographic and clinical information will be obtained from participants’ electronic health records at T0 and will include their date of birth, gender, ethnicity, country of birth, highest level of educational attainment, highest level of employment, current employment status, primary mental health file diagnosis, co-occurring mental health file diagnoses, date of first contact with mental health services, number of listed episodes of outpatient mental health care, and number of psychiatric admissions.

#### Primary Outcome

The primary outcome is personal recovery, as measured by the 15-item version of the Questionnaire about the Process of Recovery (QPR) [40,41]. The 15-item QPR has good internal consistency (QPR total α=.89), fair to good test-retest reliability (QPR total intracluster correlation coefficient [ICC]=0.74), moderate sensitivity to change, and adequate convergent validity [42]. The QPR is a measure that assesses the 5 core processes of personal recovery that comprise the “Connectedness; Hope and optimism about the future; Identity; Meaning in life; and Empowerment” (CHIME) framework [43], which is currently the strongest conceptualization of personal recovery in serious mental illness. The Recovery ACT group program seeks to support change in these dimensions for participants.

#### Secondary Outcomes

The secondary outcomes are well-being (Clinical Outcomes in Routine Evaluation 10 [CORE-10]) [44] and 4 psychological flexibility process variables, namely committed action (the Valuing Questionnaire’s Progress scale comprised of items 3, 4, 5, 7, and 9) [45], mindfulness (Southampton Mindfulness Questionnaire) [46], cognitive defusion (Cognitive Fusion Questionnaire) [47], and experiential avoidance (Brief Experiential Avoidance Questionnaire) [48].

#### Other Clinical Outcomes

A facilitator will rate illness severity at enrollment and before the start of the group using the “Clinical Global Impression–Severity” scale and will rate illness improvement at the end of the group and at the booster session using the “Clinical Global Impression–Improvement” scale [49,50].

#### Consumer and Facilitator Feedback

Permission will be sought from participants to use their responses to routine qualitative feedback questions for evaluation purposes. These questions ask about their hopes and expectations for the group (at T0), helpful and unhelpful aspects of the group (at T2 and T3), requests for remaining sessions (at T2), and reflection on any perceived changes attributed to the group (at T3 and T4). The method of collecting these routine feedback responses is dependent on the site. The baseline feedback questions (at T0) are typically asked and recorded by a facilitator during the “Information and Engagement” session; the feedback questions at other time points are typically written responses by participants. Qualitative data about the group will be sought from facilitators who consent to participate in a semistructured interview after completing a group. The qualitative interview will assess the feasibility of the group, its evaluation in routine practice, and perceptions of the group program’s effectiveness.

In addition, during the initial consent process, participants will be asked for written consent to contact them in the future to invite them to participate in a follow-up study involving a qualitative interview exploring their experience of participating in the group.

### Statistical Methods and Sample Size

We intend to use an intention-to-treat sample to test a univariate latent growth curve model of the primary outcome from before the start of the waitlist period to the start of the group to the booster session in order to compare the trajectory over the waitlist period to the trajectory over the active treatment period. We selected this method of analysis to strengthen our estimations of associations due to the limitations of the nonrandomized study design chosen as the most realistic option for an evaluation of a program already in routine practice [51]. A minimum sample size of 90 is required to conduct the proposed growth curve structural equation model (5 observed variables and 2 latent variables) with an anticipated medium effect size (0.3), α set at .05, and power (1–β) at .80. Univariate latent growth curve models for each of the 5 secondary outcomes will also be tested. If there are sufficient data, we will test whether the trajectory of the 4 psychological flexibility process variables from the start of the group to the midpoint session (T1 to T2) is significantly associated with the trajectory of personal recovery (T2 to T4) using multivariate growth models. We do not predict a priori any differences associated with the study site. We intend to calculate the ICC after the data are collected to determine the relatedness of the data collected at different study sites.

#### Randomization and Blinding

This study is a nonrandomized (unblinded) trial that uses a single group, waitlist control, and a quasiexperimental design. All participants serve as their own control. The data for all participants on change in outcomes over the waitlist period (no active intervention) will be used to compare with the data on change in outcomes during the active intervention. The planned statistical methods will strengthen our conclusions. The risk of researcher bias is lessened by the self-report format of the primary and secondary outcomes and the opportunity for participants to submit questionnaires in a sealed envelope.

#### Data Collection and Management

The data will be collected using deidentified hardcopies of all evaluation documents (quantitative questionnaires completed by participants and facilitators, copies of qualitative feedback forms, and demographic and clinical information forms), and other trial documents (attendance records, fidelity logs, and recruitment logs). Facilitators will assist participants with questionnaire completion if needed. As facilitators will serve a dual role as clinical service providers and study personnel, participants will be provided a sealable envelope to return deidentified self-report questionnaires that will be reviewed by study personnel other than their facilitators if sealed (either at the site or by the study coordinator). If a participant does not attend a group session when data are collected, facilitators will attempt to schedule an appointment to complete study measures.

Deidentified data from hard copy evaluation documents will be entered into a password-protected electronic file maintained on secure computers by the study coordinator. A coding sheet with identifiable participant information will be saved as a password-protected electronic file on the sponsor site’s server. Hard copies of consent forms and study documents will be kept in a locked filing cabinet at the Academic Psychology Unit located at the sponsor’s site.

#### Data Monitoring and Adverse Event Reporting

Anticipated risks to participant safety due to participation in the evaluation and the group are deemed minimal based on pilot study data [35]. Therefore, there is no data monitoring committee, and there are no planned interim analyses to identify risks. Adverse events will be monitored by facilitators, site principal investigators, the coordinating principal investigator, and the Interagency Investigator Committee. An adverse event form and guideline are used.

An Interagency Investigator Committee led by the coordinating principal investigator meets quarterly to oversee the trial’s progress, monitor any adverse events or serious adverse events related to the procedures or the group program, and discuss any necessary protocol modifications. A study coordinator is responsible for liaising with the site principal investigators, who are responsible for the evaluation at each site. In addition to the published Recovery ACT group program manual, evaluation procedure manuals were created to ensure that the protocol is adhered to by study personnel at all sites and over the course of the multiyear study.

### Ethical Considerations

Melbourne Health’s Human Research Ethics Committee (HREC/51498/MH-2019) provided ethics approval for this study. All methods will be performed in accordance with the relevant guidelines and regulations, and written, informed consent will be obtained from participants. The results of the main aim of the study will be published in a peer-reviewed scientific journal and submitted for presentation at clinical and scientific meetings and conferences. The coordinating principal investigator will oversee the publication and presentation of study results, including decisions about authorship. Participants are asked whether they would like to be informed of the main results of the study. Those who express interest and provide contact details will be provided with this information when it is available. Participants who complete quantitative outcome measures will be compensated with a US $6.54 department store voucher at each time point.

## Results

Trial enrollment began in September 2019 and was originally planned to continue until at least March 2022. In March 2020, the Victorian Government in Australia introduced public health directions in response to the COVID-19 pandemic that derailed study progress as in-person groups were not permitted for over 20 months at some sites. While study recruitment and follow-up study procedures were broadened to allow for the use of telehealth, the group program format (in-person) was not changed as it would introduce significant variability to the study outcomes. As there remains notable interest from all stakeholders (investigators, facilitators, managers, and consumers), the study will continue to accept enrollments until at least 2024. As of the submission of the manuscript, 93 participants consented to the evaluation, 65 completed T1 measures, and 40 had a complete dataset for the proposed analyses. No data analysis has been conducted. Due to the uncertainty of the trial’s viability due to the public health directions during the COVID-19 pandemic, the protocol’s publication was delayed. The current protocol version is 8.0 (issue date: July 14, 2021).

## Discussion

To our knowledge, this is the first trial in Australia evaluating the effectiveness of an ACT for psychosis group program on personal recovery outcomes. Although limited by the single-group design, its strength lies in the opportunity to evaluate effectiveness in routine practice. External validity is strengthened by the practice settings being spread across 4 public mental health networks in a large city, serving communities with a range of sociodemographic characteristics.

A randomized design was not feasible as the program is offered in routine practice [51], thus limiting causal conclusions. However, the quasiexperimental design includes a waitlist period that provides a baseline comparison to evaluate the effectiveness of the group program and can also allow for process analyses if sufficient data are collected. A further enhancement is a proposed qualitative follow-up study of the experience of the group program, intended to inform program development and assist in the interpretation of quantitative results.

A feature of note is the clinician-initiated origin of the evaluation and the forging of a partnership between clinicians and academics during a previous pilot evaluation that then led to the co-design and collaborative implementation of the trial [34]. This clinician-researcher partnership is likely to not only ensure the practical applicability of program modifications and the feasibility of study procedures, but may also lay the foundations for more rapid implementation into practice should the results warrant it. Nonetheless, as an unfunded study, the project is subject to significant risk of noncompletion as it relies on the participating mental health services supporting facilitators to undertake some evaluation tasks, services continuing to provide the group program over the proposed study period, and the support of the sponsor site to provide a study coordinator.

This study exemplifies an effectiveness-implementation hybrid trial [52]. If successful, the trial will further support psychosocial interventions to improve personal recovery among consumers who experience persisting psychotic symptoms.

## Appendix

Multimedia Appendix 1 Standard Protocol Items: Recommendations for Interventional Trials (SPIRIT) 2013 Checklist (36).

## Abbreviations

ACT: Acceptance and Commitment Therapy
CBTp: cognitive behavioral therapy for psychosis
CHIME: Connectedness; Hope and optimism about the future; Identity; Meaning in life; and Empowerment
CONSORT: Consolidated Standards of Reporting Trials
CORE-10: Clinical Outcomes in Routine Evaluation 10
ICC: intracluster correlation coefficient
QPR: Questionnaire about the Process of Recovery
SMART: specific, meaningful, adaptive, realistic, and time-framed
SPIRIT: Standard Protocol Items: Recommendations for Interventional Trials

## Data Availability

The data sets generated during or analyzed during this study are not publicly available as the authors do not have research participants’ permission to share data.
