# Supplementary material for: Group Acceptance and Commitment Therapy for Recovery From Psychosis: Protocol for a Single-Group Waitlist Trial
Source: JMIR Res Protoc. 2024 Mar 18;13:e49849. doi: 10.2196/49849 (PMC10985603; doi:10.2196/49849)
Supplement: Multimedia Appendix 1 [file resprot_v13i1e49849_app1.pdf]

Additional File 1.

Standard Protocol Items: Recommendations for Interventional Trials (SPIRIT) 2013 Checklist (36)[i].

		Reporting Item	Page and Line Number	Reason if not applicable
**Administrative information**				
Title	#1	Descriptive title identifying the study design, population, interventions, and, if applicable, trial acronym	Page 1, Line Number (LN) 4	
Trial registration	#2a	Trial identifier and registry name. If not yet registered, name of intended registry	Page 3, LN 3-4; Page 4 LN 1	
Trial registration: data set	#2b	All items from the World Health Organization Trial Registration Data Set	Page 9, LN1 9; Page 31, Table 1	
Protocol version	#3	Date and version identifier	Page 4, LN1; Page 17, LN 17-18	
Funding	#4	Sources and types of financial, material, and other support	Page 4, LN1; Page 12, LN 1-2; Page 19, LN 18-22	
Roles and responsibilities: contributorship	#5a	Names, affiliations, and roles of protocol contributors	Page 1, LN 8-26; Page 19 LN 23 to Page 20 LN 3	
Roles and responsibilities: sponsor contact information	#5b	Name and contact information for the trial sponsor	Page 4, LN1	

Roles and responsibilities: sponsor and funder	#5c	Role of study sponsor and funders, if any, in study design; collection, management, analysis, and interpretation of data; writing of the report; and the decision to submit the report for publication, including whether they will have ultimate authority over any of these activities	Page 4, LN 1	Not applicable as this is an investigator-initiated trial and is unfunded.
Roles and responsibilities: committees	#5d	Composition, roles, and responsibilities of the coordinating centre, steering committee, endpoint adjudication committee, data management team, and other individuals or groups overseeing the trial, if applicable (see Item 21a for data monitoring committee)	Page 16, LN 7-21	
**Introduction**				
Background and rationale	#6a	Description of research question and justification for undertaking the trial, including summary of relevant studies (published and unpublished) examining benefits and harms for each intervention	Page 5 LN1 to Page 7 LN 10	
Background and rationale: choice of comparators	#6b	Explanation for choice of comparators	Page 7, LN 12-17	
Objectives	#7	Specific objectives or hypotheses	Page 7 LN 12 to Page 8 LN 6	
Trial design	#8	Description of trial design including type of trial (eg, parallel group, crossover, factorial, single group), allocation ratio, and framework (eg,	Page 8 LN 24 to Page 9 LN1	

		superiority, equivalence, non-inferiority, exploratory)		
**Methods: Participants, interventions, and outcomes**				
Study setting	#9	Description of study settings (eg, community clinic, academic hospital) and list of countries where data will be collected. Reference to where list of study sites can be obtained	Page 8 LN 9-16	
Eligibility criteria	#10	Inclusion and exclusion criteria for participants. If applicable, eligibility criteria for study centres and individuals who will perform the interventions (eg, surgeons, psychotherapists)	Page 8 LN 17-22	
Interventions: description	#11a	Interventions for each group with sufficient detail to allow replication, including how and when they will be administered	Page 10 LN 19 to Page 11 LN 12	
Interventions: modifications	#11b	Criteria for discontinuing or modifying allocated interventions for a given trial participant (eg, drug dose change in response to harms, participant request, or improving / worsening disease)	Page 11 LN 13-17	
Interventions: adherence	#11c	Strategies to improve adherence to intervention protocols, and any procedures for monitoring adherence (eg, drug tablet return; laboratory tests)	Page 11 LN 11-12, Page 15 LN 18-19	
Interventions: concomitant care	#11d	Relevant concomitant care and interventions that are permitted or prohibited during the trial	Page 12 LN 1-4	

Outcomes	#12	Primary, secondary, and other outcomes, including the specific measurement variable (eg, systolic blood pressure), analysis metric (eg, change from baseline, final value, time to event), method of aggregation (eg, median, proportion), and time point for each outcome. Explanation of the clinical relevance of chosen efficacy and harm outcomes is strongly recommended	Page 12 LN 10 to Page 14 LN 13; Page 29 LN 1; Page 30 LN 1	
Participant timeline	#13	Time schedule of enrolment, interventions (including any run-ins and washouts), assessments, and visits for participants. A schematic diagram is highly recommended (see Figure)	Page 29 LN1; Page 30 LN 3	
Sample size	#14	Estimated number of participants needed to achieve study objectives and how it was determined, including clinical and statistical assumptions supporting any sample size calculations	Page 14 LN 21-23	
Recruitment	#15	Strategies for achieving adequate participant enrolment to reach target sample size	Page 9 LN 21 to Page 10 LN 18	
**Methods: Assignment of interventions (for controlled trials)**				
Allocation: sequence generation	#16a	Method of generating the allocation sequence (eg, computer-generated random numbers), and list of any factors for stratification. To reduce predictability of a random sequence, details of any planned restriction (eg, blocking) should be provided in a separate document that		Not applicable as it is a non-randomized trial.

		is unavailable to those who enrol participants or assign interventions		
Allocation concealment mechanism	#16b	Mechanism of implementing the allocation sequence (eg, central telephone; sequentially numbered, opaque, sealed envelopes), describing any steps to conceal the sequence until interventions are assigned		Not applicable as it is an open trial and masking is not used.
Allocation: implementation	#16c	Who will generate the allocation sequence, who will enrol participants, and who will assign participants to interventions		Not applicable as it is a non-randomized trial.
Blinding (masking)	#17a	Who will be blinded after assignment to interventions (eg, trial participants, care providers, outcome assessors, data analysts), and how		Not applicable as it is an open trial and masking is not used.
Blinding (masking): emergency unblinding	#17b	If blinded, circumstances under which unblinding is permissible, and procedure for revealing a participant's allocated intervention during the trial		Not applicable as it is an open trial and masking is not used.
**Methods: Data collection, management, and analysis**				
Data collection plan	#18a	Plans for assessment and collection of outcome, baseline, and other trial data, including any related processes to promote data quality (eg, duplicate measurements, training of assessors) and a description of study instruments (eg, questionnaires, laboratory tests) along with their reliability and validity, if known. Reference	Page 13 LN 3 to Page 14 LN 13; Page 15 LN 14 to Page 16 LN 6	

		to where data collection forms can be found, if not in the protocol		
Data collection plan: retention	#18b	Plans to promote participant retention and complete follow-up, including list of any outcome data to be collected for participants who discontinue or deviate from intervention protocols	Page 15 LN 22-24	
Data management	#19	Plans for data entry, coding, security, and storage, including any related processes to promote data quality (eg, double data entry; range checks for data values). Reference to where details of data management procedures can be found, if not in the protocol	Page 15 LN 14 to Page 15 LN 16	
Statistics: outcomes	#20a	Statistical methods for analysing primary and secondary outcomes. Reference to where other details of the statistical analysis plan can be found, if not in the protocol	Page 14 LN 14 to Page 15 LN 5	
Statistics: additional analyses	#20b	Methods for any additional analyses (eg, subgroup and adjusted analyses)	Page 14 LN 24 to Page 15 to LN 5	
Statistics: analysis population and missing data	#20c	Definition of analysis population relating to protocol non-adherence (eg, as randomised analysis), and any statistical methods to handle missing data (eg, multiple imputation)	Page 15 LN 6-13	
**Methods: Monitoring**				
Data monitoring: formal committee	#21a	Composition of data monitoring committee (DMC); summary of its role and reporting	Page 16 LN 7-13	

		structure; statement of whether it is independent from the sponsor and competing interests; and reference to where further details about its charter can be found, if not in the protocol. Alternatively, an explanation of why a DMC is not needed		
Data monitoring: interim analysis	#21b	Description of any interim analyses and stopping guidelines, including who will have access to these interim results and make the final decision to terminate the trial	Page 16 LN 7-13	
Harms	#22	Plans for collecting, assessing, reporting, and managing solicited and spontaneously reported adverse events and other unintended effects of trial interventions or trial conduct	Page 16, LN 10-13	
Auditing	#23	Frequency and procedures for auditing trial conduct, if any, and whether the process will be independent from investigators and the sponsor		Not applicable as no auditing procedures are in place.
**Ethics and dissemination**				
Research ethics approval	#24	Plans for seeking research ethics committee / institutional review board (REC / IRB) approval	Page 16, LN 23-24	
Protocol amendments	#25	Plans for communicating important protocol modifications (eg, changes to eligibility criteria, outcomes, analyses) to relevant parties (eg, investigators, REC / IRBs, trial participants, trial registries, journals, regulators)	Page 16, LN 14-21	

Consent or assent	#26a	Who will obtain informed consent or assent from potential trial participants or authorised surrogates, and how (see Item 32)	Page 10 LN 5-11	
Consent or assent: ancillary studies	#26b	Additional consent provisions for collection and use of participant data and biological specimens in ancillary studies, if applicable	Page 10 LN 15-18	
Confidentiality	#27	How personal information about potential and enrolled participants will be collected, shared, and maintained in order to protect confidentiality before, during, and after the trial	Page 15 LN 14 to Page 16 LN 6	
Declaration of interests	#28	Financial and other competing interests for principal investigators for the overall trial and each study site	Page 19 LN 8-9	
Data access	#29	Statement of who will have access to the final trial dataset, and disclosure of contractual agreements that limit such access for investigators	Page 19 LN 15-17	
Ancillary and post trial care	#30	Provisions, if any, for ancillary and post-trial care, and for compensation to those who suffer harm from trial participation	Page 12 LN 3-9	
Dissemination policy: trial results	#31a	Plans for investigators and sponsor to communicate trial results to participants, healthcare professionals, the public, and other relevant groups (eg, via publication, reporting in results databases, or other data sharing arrangements), including any publication restrictions	Page 16 LN 24 to Page 17 LN 5	

Dissemination policy: authorship	#31b	Authorship eligibility guidelines and any intended use of professional writers	Page 17 LN 1-2	
Dissemination policy: reproducible research	#31c	Plans, if any, for granting public access to the full protocol, participant-level dataset, and statistical code	Page 19 LN 15-17	
**Appendices**				
Informed consent materials	#32	Model consent form and other related documentation given to participants and authorised surrogates		Not included.
Biological specimens	#33	Plans for collection, laboratory evaluation, and storage of biological specimens for genetic or molecular analysis in the current trial and for future use in ancillary studies, if applicable		Not applicable as no biological specimens are collected.
